# A Revised Design for Microarray Experiments to Account for Experimental Noise and Uncertainty of Probe Response

**DOI:** 10.1371/journal.pone.0091295

**Published:** 2014-03-11

**Authors:** Alex E. Pozhitkov, Peter A. Noble, Jarosław Bryk, Diethard Tautz

**Affiliations:** 1 Max-Planck-Institut für Evolutionsbiologie, Plön, Germany; 2 Department of Periodontics, School of Dentistry, University of Washington, Seattle, Washington, United States of America; 3 Ph.D Microbiology Program, Department of Biological Sciences, Alabama State University, Montgomery, Alabama, United States of America; 4 National Centre for Biotechnology Education, University of Reading, Reading, United Kingdom; Technische Universität Dresden, Medical Faculty, Germany

## Abstract

**Background:**

Although microarrays are analysis tools in biomedical research, they are known to yield noisy output that usually requires experimental confirmation. To tackle this problem, many studies have developed rules for optimizing probe design and devised complex statistical tools to analyze the output. However, less emphasis has been placed on systematically identifying the noise component as part of the experimental procedure. One source of noise is the variance in probe binding, which can be assessed by replicating array probes. The second source is poor probe performance, which can be assessed by calibrating the array based on a dilution series of target molecules. Using model experiments for copy number variation and gene expression measurements, we investigate here a revised design for microarray experiments that addresses both of these sources of variance.

**Results:**

Two custom arrays were used to evaluate the revised design: one based on 25 mer probes from an Affymetrix design and the other based on 60 mer probes from an Agilent design. To assess experimental variance in probe binding, all probes were replicated ten times. To assess probe performance, the probes were calibrated using a dilution series of target molecules and the signal response was fitted to an adsorption model. We found that significant variance of the signal could be controlled by averaging across probes and removing probes that are nonresponsive or poorly responsive in the calibration experiment. Taking this into account, one can obtain a more reliable signal with the added option of obtaining absolute rather than relative measurements.

**Conclusion:**

The assessment of technical variance within the experiments, combined with the calibration of probes allows to remove poorly responding probes and yields more reliable signals for the remaining ones. Once an array is properly calibrated, absolute quantification of signals becomes straight forward, alleviating the need for normalization and reference hybridizations.

## Introduction

Microarrays have been extensively used for examining gene expression and for detecting single nucleotide polymorphisms (SNPs) or copy number variations (CNVs) in genomic DNA [1], [2]. Yet, despite the general use of this technology, uncertainty remains in the interpretation of the array output. For example, several studies have shown that about 20 to 30% of expressed genes are identified as either up- or down-regulated solely depending on the algorithm used [3]–[5]. In an experiment assessing expression differences between mouse populations, we found correlation coefficients of less than 0.7 between an Affymetrix (25 mer) and an Agilent (60 mer) platform, although identical RNA samples were used [6]. Hence, it is currently routinely required to apply additional experimental tests, such as quantitative PCR, to verify results obtained from microarrays.

We argue that one reason for the uncertainty in the interpretation of the array output is insufficient measurement of experimental noise in current protocols. In the first generation array platforms (spotted arrays), the noise problem was mostly due to uneven surfaces of arrays and variability between arrays (e.g., ref [7]). This problem is now largely solved, partly because manufacturing of arrays has significantly improved and because internal quality checks are routinely implemented to account for this problem. Still, any quantitative measurement is associated with measurement errors and even for a perfectly manufactured array, a determination of this error is expected to raise the statistical confidence in the measurement. However, an assessment of this measurement error has so far not been implemented in the experimental procedures of microarray hybridization.

Another problem for the optimal design of arrays is the uncertainty of probe binding behavior. Although many parameters have been identified that affect probe binding behavior [8]–[13], it remains a challenge to design arrays in a way that makes probe binding behavior predictable. In high density arrays, such as Affymetrix, this problem is partly solved by averaging results across multiple different probes for the same target (e.g., Affymetrix probe sets, www.affymetrix.com). Although this yields a major improvement in signal reliability, it is nonetheless still an inherently noisy procedure, since poorly responding probes may influence the signal in unpredictable ways. The alternative is validation and calibration of probes and we explore this here.

Our revised design for microarray experiments includes an estimate and control of experimental noise, as well as calibration of probes with a biological sample. Specifically, the calibration of probes allows one to identify poorly responding probes and subsequently remove them from the analysis. In addition, calibration allows one to directly determine target concentrations in biological samples from signal intensity, without the need to use reference hybridizations. To show that these procedures can improve the accuracy of quantitative measurements using microarrays, we use two types of test arrays: one with short (25 mer) probes and another with long (60 mer) probes. Using test hybridization and adjusted statistical procedures, we show that a major improvement of signal reliability can indeed be obtained.

## Materials and Methods

All animal work followed the legal requirements, was registered under number V312-72241.123-34 (97-8/07) and approved by the ethics comission of the Ministerium für Landwirtschaft, Umwelt und ländliche Räume, Kiel (Germany) on 27. 12. 2007.

### Array experiments

**Figure 1:**
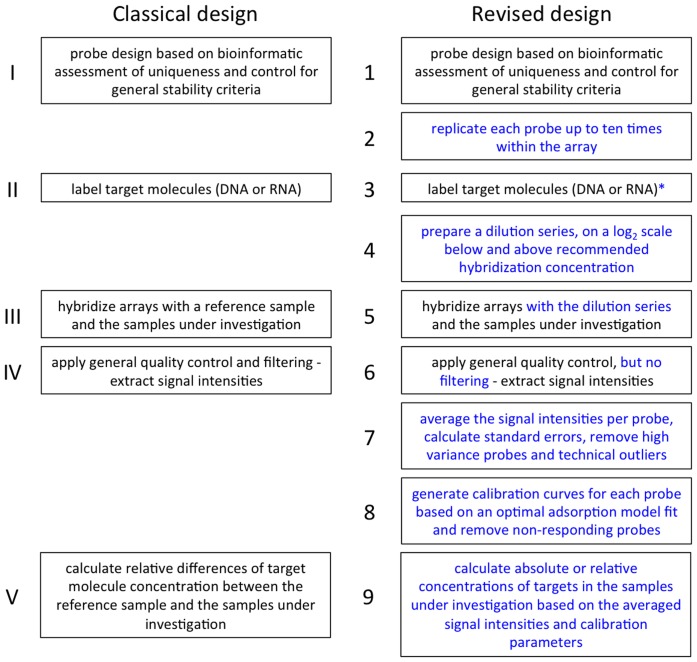
Comparison of the classical and the revised experimental design. Workflows are from top to bottom and equivalent stages are set next to each other. New steps are in blue type face. Both workflows represent only general schemes and further variations are possible. For example, we discuss also an additional step for the target labeling procedure in the text (denoted by an asterisk in step 3).

The general workflow of the revised design is depicted in Figure 1. To test this in model experiments, two custom arrays were designed and manufactured by Agilent (Santa Clara, Calif.). The first array (henceforth designated “25 mer array”) consisted of 5,912 25 mer probes, each replicated ten times on the array. The probes represented genes from the mouse X-chromosome and the probe sequences were taken from the Affymetrix “GeneChip Mouse Genome 430 2.0 Array”. The second array (henceforth designated as “60 mer array”) consisted of 4,614 60 mer probes, each replicated ten times on the array. They were designed by Agilent to trace regions of structural variation, such as copy-number variation (CNV) in the mouse genome. All probes in both arrays were placed in random locations to allow the determination of binding variance in an unbiased way. The 25 mer array was used to test the general utility of the approach for DNA and RNA hybridizations. The 60 mer array was used to compare the performance of the Agilent standard procedure for CNV discovery to our protocol.

**Figure 2:**
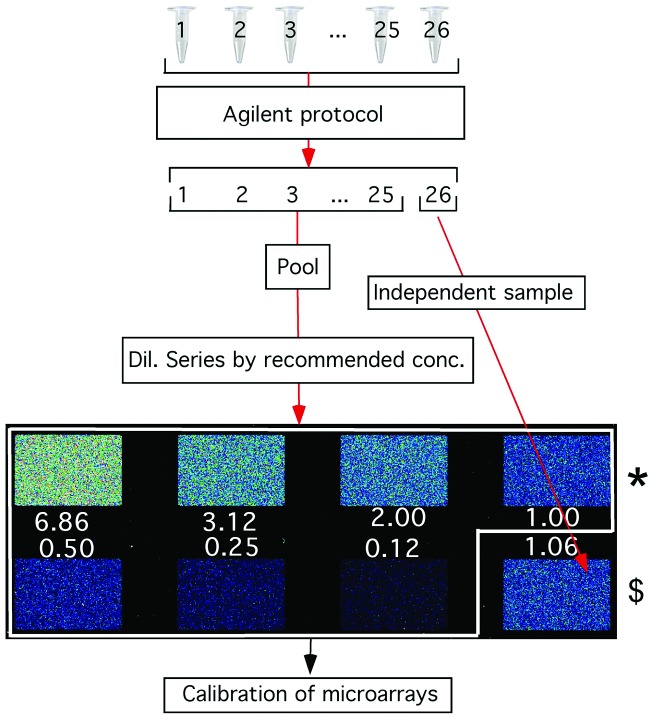
Outline of the experimental design for recording a dose-response curve for each probe on an 8-plex Agilent microarray. The dilution series was created by pooling the labeled samples, serially diluting the pool, and hybridizing each diluted sample to an independent array. The arrays within the white box were used to calibrate the probes. One array marked with an asterisk (*) was used as a ‘reference’. The independent ‘test’ array ($) is also shown. Numerical values indicate the target concentration in folds of the recommended concentration.

Genomic DNA (gDNA) and RNA was labeled according to the manufacturer's recommended protocol (Agilent). For the gDNA and RNA dilution series experiments (Figure 2), several samples of the recommended concentration were independently labeled. For gDNA, the labeled products were pooled together, precipitated with sodium acetate, and the resulting pellet was dissolved in Tris-EDTA buffer (10 mM Tris, 1 mM EDTA, pH 8.0). Then, the concentration of the DNA was measured with NanoDrop (Thermo Scientific Inc.), and a dilution series was prepared. For RNA, the yield was sufficient to make a dilution series by mixing several 5 µl aliquots of the independently labeled products and diluting the mix accordingly. Hybridization was conducted in the Agilent hybridization buffer at 48°C for approx. 17 h followed by recommended washing.

**Probe calibration:** Depending on the probe, it was possible to model the isotherms using the adsorption equations proposed by Freundlich [14] or Langmuir [15]–[23]. We have devised an automated algorithm to use the better fitting model for each probe (see software in File S2) and applied it accordingly. The Langmuir equations is:
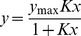
(1)where *y* is signal intensity; *x* – concentration; *K* – binding constant; *y_max_* – saturation level.

The Freundlich equation is:
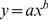
(2)where *y* is signal intensity; *x* – concentration; *a, b –* empirical parameters.

### Error calculation

The purpose of determining the relative error of the mean signal intensity *Err(y)* is to determine the relative error associated with the calculated concentration, *Err(x)*. If the *Err(x)* is less than the acceptable level (e.g., 20%) than the calculated target concentration can be trusted. Otherwise the respective probe was removed from the further analysis. The *Err(y)* is calculated according to the sampling distribution [24] as standard deviation of *y* divided by the square root of the number of replicates and divided by the average of *y*. *Err(x)* is dependent on the model (see Equation 5 and Equation 6).

**Error calculation for the Freundlich equation:**An assessment of the error for the calculated concentration from the calibration curves can be achieved as follows. Given the form of the calibration curve (Equation 1),
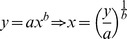
(3)one determines the relative error of *x* upon the error of *y*, by finding the differentials according to the error propagation theory [25]. Assuming small uncertainties of the parameters *a* and *b*, which can be ensured by selecting calibration curves with a high goodness of fit (see below), the differentials are given by Equation 4. 
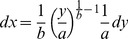
(4)


The differentials are equivalent to standard deviations [25]. Dividing both sides of the Equation 4 by the Equation 2 yields relative errors of *x* and *y*:
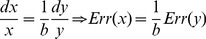
(5)


**Error calculation for the Langmuir equation:**From the Langmuir equation, *x* (i.e., concentration) is obtained as follows:
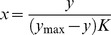



The error for calculated concentration is found according to the error propagation theorem [25].



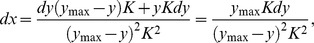
Where *dx* and *dy* are standard deviations of *x* and *y* respectively.The relative error is as follows:
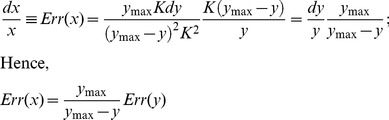
(6)


### Data analysis

The data were stored and analyzed in an MS SQL database. We wrote three C++ programs to analyze the data for users. The program executables, documentation, the programs' code as well as an example dataset are provided in File S2) or can be downloaded at: http://web.evolbio.mpg.de/~alexander.pozhitkov/microarray123/.

The probe lists and microarray data were submitted to datadryad.org and are available under doi:10.5061/dryad.57ms3.

## Results

Our study was initially motivated by an attempt to use the Affymetrix mouse genome diversity arrays [26] to assess CNV between mouse strains and populations. While these arrays were primarily designed to screen for SNPs, they contain also a set of ∼1.8 million probes that were specifically designed to represent non-polymorphic regions of the genome for CNV detection [26]. Each of these probes was designed by state of the art principles and is present as a sense and an antisense version on the array. One would expect that corresponding sense and antisense probes should provide the same signal when hybridized to genomic DNA, since in their hybridized state (i.e., double helix), they should be equivalent with respect to base composition and secondary structure.

We analyzed the range of signal intensities of these invariant probes and found that it spans over almost four orders of magnitude, i.e. deviate significantly from an expectation of similar hybridization efficiency. We assessed whether differences in GC composition or Gibbs free energy parameters could explain this, but neither parameter was significantly correlated with the signal intensities of the probes (Figure S1A and B in File S1). Moreover, plotting the relationship of signal intensities between the corresponding sense and antisense probes revealed very high variation (Figure S1C in File S1), although there was an overall correlation (R^2^ = 0.37). Still, this variation suggests that it is not directly possible to predict signal intensity of an antisense probe given the intensity of a sense version of the same probe.

To investigate this further, we compared the experimentally measured melting temperatures of five sense-antisense probe pairs on the array and in solution (Table S1 in File S1). As predicted by theory (Figure S2A in File S1), we found a good correlation of melting behavior between sense and antisense probes in solution (R^2^ = 0.96), but the melting temperatures in solution did not correlate with the signal intensities of the probes on the array (Figure S2B in File S1). Although these analyses and experiments have only a preliminary character, they suggest that the physicochemical hybridization parameters determined in solution differ from those on surfaces. Getting deeper insights into this is an active field of research [27] and it is hoped that it will be better understood at some point. However, until these problems are solved, we decided here to devise an empirical ad hoc procedure to address the problems of limited predictability of hybridization behavior. At the same time, we introduce a step to control for the unavoidable noise inherent in any measurement, including hybridization reactions.

### Sources of variance

We conjectured that there are two major sources that produce uncontrolled variance. The first source is the experimental variance of signal generation, i.e., hybridization and washing, and the second is the poorly known probe responsiveness (as shown above). A further source of error may be the variance in sample preparation (e.g., [30], which we address as well. All these sources can be investigated - and thus controlled - by appropriately designed experiments.

Experimental variance of signal generation can be measured by replicating identical probes on the same array. Assuming homogeneous hybridization conditions across the array (which is mostly the case for today's commercial hybridization systems), one should expect that the variances of the signals coming from these identical probes are a direct measure of technical noise associated with the hybridization itself.

Probe responsiveness can be empirically assessed by hybridizing an array with a dilution series of a given mix of targets, e.g., genomic DNA (gDNA). The individual probe hybridization isotherms can then be obtained by plotting the relationship between the diluents (target concentrations) and signal intensities. Their shape will reveal if the isotherms follow a predictable dose-response relationship and thus can be used for quality filtering, e.g., to remove non-responsive probes.

Below, we assess a revised experimental design, outlined in Figure 1, that takes care of the two major sources of variance identified above and we present model experiments that verify this conjecture.

### Measurement error

**Figure 3:**
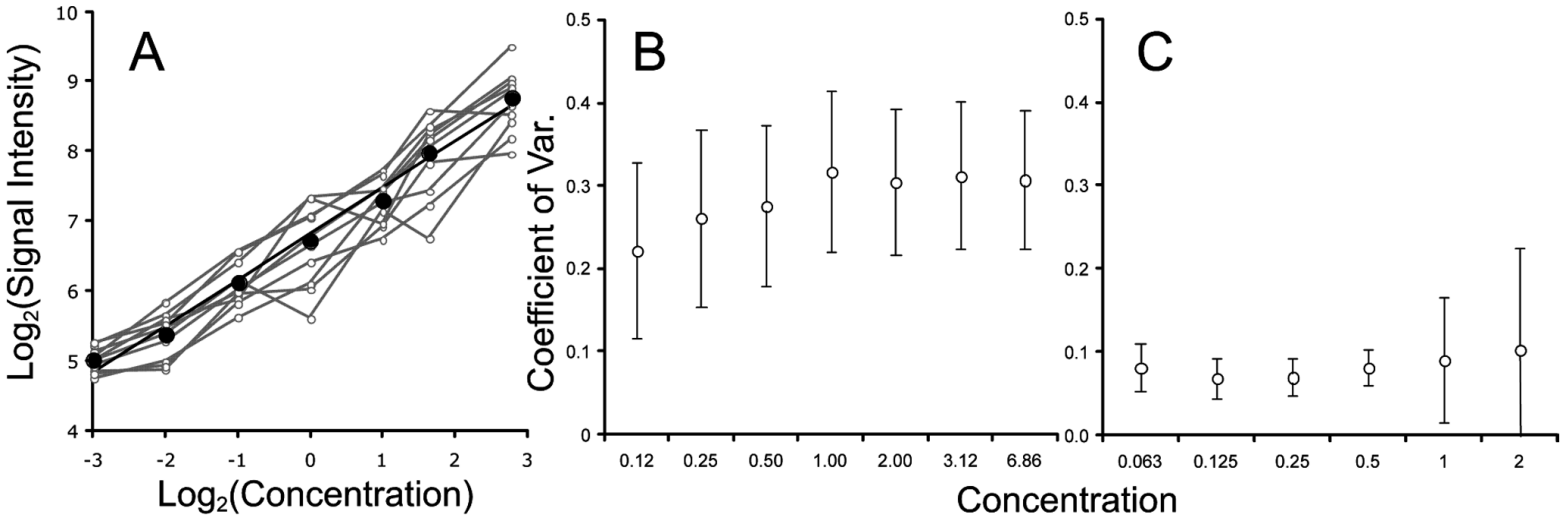
Signal variances between replicated probes. (A) Typical Agilent array isotherms obtained using a dilution series of genomic mouse DNA, BL6 strain for a single probe and its replicates. Raw data (gray) and predicted isotherm based on the average signal intensity (black). (B) Mean and standard deviation of the coefficient of variation (CV) across all probes at each concentration for the 25 mer array, (C) same for the 60 mer array.

We assessed the extent of measurement error associated with hybridization and probe binding that is inherent in the standard microarray procedure by comparing the signals from ten replicated probes within each array. The arrays were hybridized with genomic DNA (gDNA) using the dilution series depicted in Figure 2. We observed indeed a general variance in the signal intensity among the 10 identical replicates of each probe. As an example, Figure 3A shows a typical case of signal intensities for a single 25 mer probe at different dilutions. In this case, we observed up to four-fold differences for identical replicates. Averaging of the signal intensities of the 10 replicates, however, yields a good fit to a power-law function (Figure 3A). Hence, the variability in signal intensity of individual probes appears to reflect the measurement error.

The majority of probes have a variation coefficient of ∼12 to 35% for the 25 mer array (Figure 3B) and ∼7 to 10% for the 60 mer array (Figure 3C), as assessed from the standard deviations of signal intensities over 10 replicates. For a few probes on each array we observed unusually strong outliers (data not shown). Inspection of these probes showed that this was always caused by technical problems (e.g., dust particles) in a single replicate. Such technical outliers can be easily identified based on the comparison with the other replicated probes and were removed.

### Calibration of probe behavior

**Figure 4:**
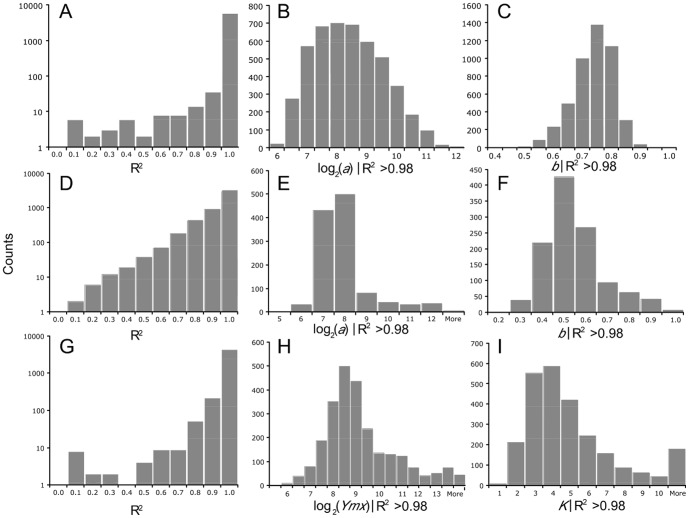
Distribution of curve fitting parameters for the isotherm models. Panels A to F, Freundlich model, Panels G to I, Langmuir model. Panels A to C, 25: Distribution of R2 across all probes. Panels B and E: Distribution of *a* for selected probes. Panel C and F: Distribution of the exponent *b* for selected probes (Equation 2). Panel H: Distribution of *y_max_* for selected probes. Panel I: Distribution of *K* for selected probes (Equation 1).

Calibration can be used to determine the probe response function (i.e., calibration curve) and thus to remove poorly responding probes. In order to obtain calibration parameters of each probe, one has to determine the respective equation parameters, e.g., R^2^, *k* and Y_max_ for the Languir equation; *a* and *b* for the Freundlich equation (see Methods). The parameter estimation is done by a linear regression of the linearized data, i.e, *x/y* vs *x* for Langmuir model and log(*y*) vs log(*x*) for Freundlich model. Probes with low R^2^ values for either equation are unlikely to be reliable for actual measurements. We suggest that probes below a cutoff of R^2^≤0.98 should be removed from further analysis, but this cutoff could be individually adjusted for each experiment. For our experiments, we found that 1092 probes (18%) fell below this cutoff for the 25 mer array and 1124 probes (24%) for the 60 mer array. For the remaining probes, we found that the majority (98%) showed a better fit with the Freundlich equation for the 25 mer array, while for the 60 mer array, 30% showed a better fit for the Freudlich equation and 70% for the Langmuir equation. We determined the parameter distributions and R^2^ values for each probe for both equations on both arrays (Figure 4). The Langmuir parameters for the 25 mer arrays are not shown in Figure 4 due to the small number of probes (<2%) that followed the Langmuir model.

In contrast to gDNA arrays (such as CNV arrays), expression arrays are usually hybridized with mRNA targets. The optimal labelling procedure for RNA involves a RNA synthesis step [28]. Because the physicochemistry of DNA:DNA hybridization differs from that of DNA:RNA [29], we expected that a calibration with a RNA target to yield different results from the calibration with the gDNA. We tested this using the 25 mer array since these probes were derived from an expression array. Averaging and calibration was done as described above for the gDNA target. Comparison of the *a* and *b* parameters of the Freundlich equation for each probe revealed little correlation between DNA and RNA (data not shown). Hence, separate calibrations are needed for RNA and DNA targets.

There is an additional problem with RNA calibration because different mRNAs occur at different concentrations in a given sample. Specifically, probe signal intensities of mRNAs expressed at low levels (i.e., at low concentrations) will fall below the background level (Figure S3 in File S1). Because of this problem, mRNA calibration should always be done in parallel to a given experiment in order to ensure appropriate representation of the mRNAs. Moreover, in contrast to the absolute calibration that is achieved for gDNA experiments, one can only determine a relative change in concentration for mRNA experiments since the concentration of the mRNAs in the calibration mix are not known.

### Assessing the improvement of signal quality

**Figure 5:**
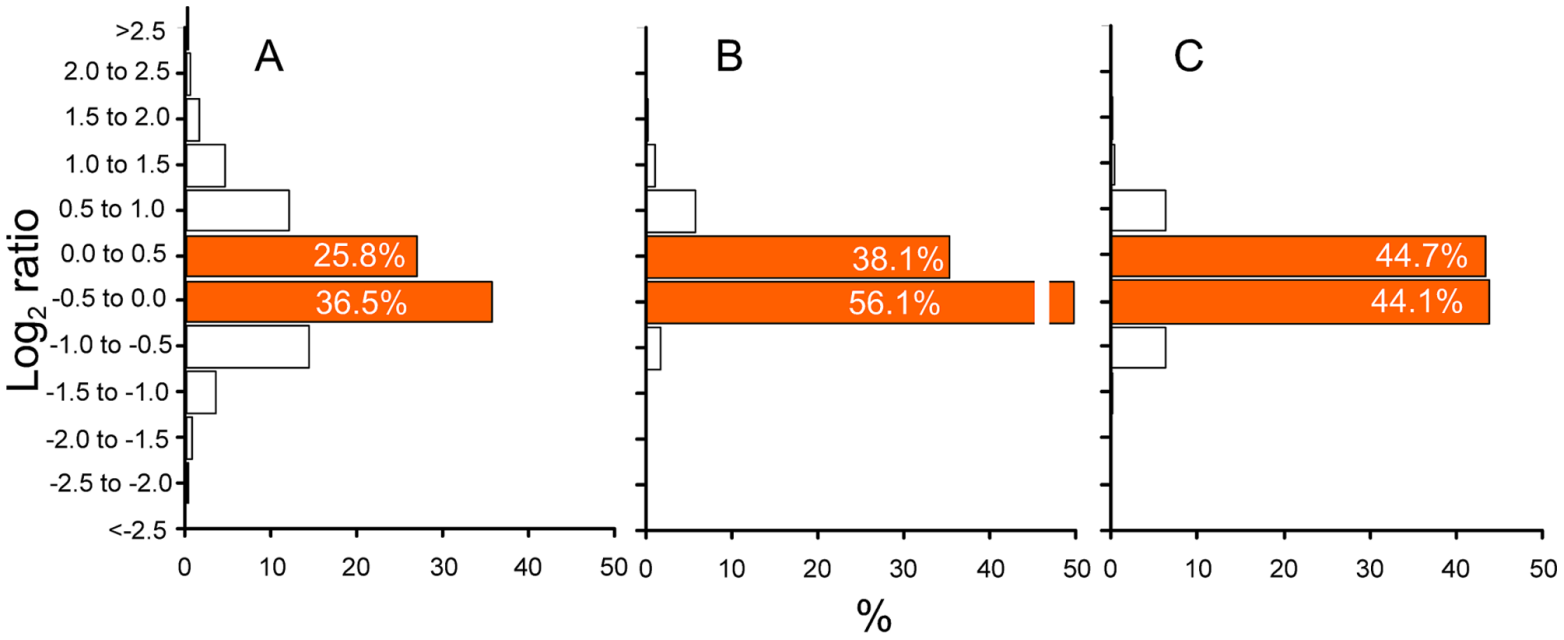
Overall assessment of noise reduction using the same DNA sample. Since the sample is compared against itself, the log_2_ ratio should be 0, i.e. all values above or below 0 are experimental noise. (A) Classical reference procedure - ratio of signal intensities between all individual probes (*n* = 5,912). (B) Averaging across probes - ratio of signal intensities from 10 averaged probes (*n* = 5,912). (C) Full revised procedure - concentration values from calibrated isotherms of all responsive probes (R^2^>0.98), a value is included only if its relative error is under 20% (*n* = 4,406).

In our first test we used identical DNA samples against each other (a reference and a test array, marked * and $ in Figure 2) in a genomic DNA (gDNA) hybridization experiment. The samples hybridized to these arrays were derived from the same DNA; therefore, no signal variation was expected and their signal intensity ratio should be equal to 0 in the log_2_ scale. Any deviation from 0 represents the noise in the experiment. Figure 5A and Figure 5B show the ratios of signal intensities of the reference and test samples for individual probes versus ten averaged replicates, respectively. For the individual probes, only 62.3% of the ratios spanned a reasonably acceptable range of log_2_ values between −0.5 to +0.5 (orange columns). For signal ratios of averaged probes, 94.2% were within this range, thus supporting the notion that probe replication on the same array can significantly improve the accuracy of the measurement. A similar improvement (88.9%) was obtained when we used calibration from averaged probes instead of ratios (Figure 5C). Although this is a bit lower than the one obtained using the ratio of the averages, the calibration method is superior because it removed probes that have non-linear behavior. The calibration approach thus results in a highly symmetric distribution and indicates that a better signal quality is obtained.

**Figure 6:**
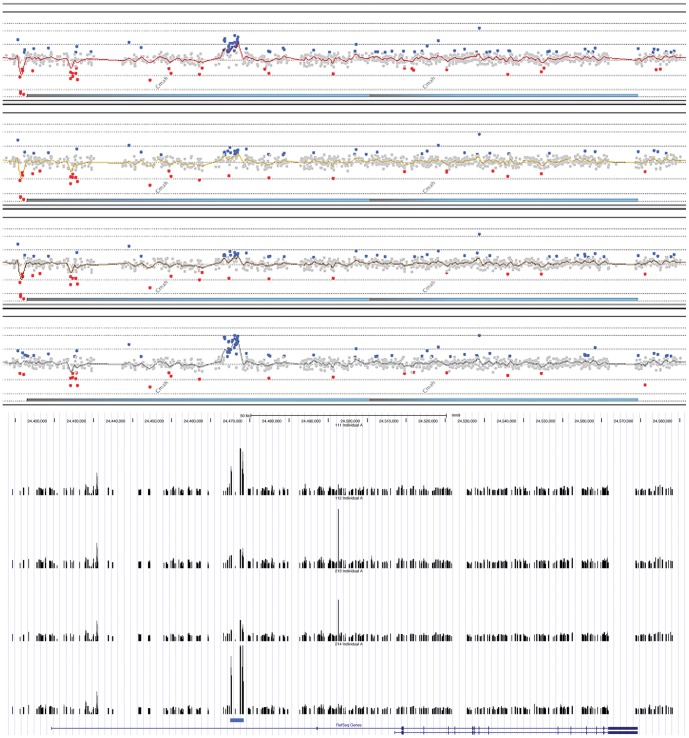
Comparison of analysis procedures for copy number variation of a gene region in mice. Four different wild type mice were analyzed, each represented as a track. Top: output from the ratio analysis implemented in the Agilent software (ratio with respect to DNA from an C57Bl/6 inbred mouse strain). The input was the concentrations derived from the ten averaged probes, but without calibration and without removal of non-responding probes. Colored dots represent values larger (blue) or smaller (red) then log_2_ = 0.5. Bottom: concentration calculations based on the full revised method, non-responding probes removed (>20% error in any of the experiments on the array). The values were normalized with respect to average intensities on the array and are displayed as custom track in the UCSC genome browser.

The second test was aimed at assessing signal improvement in an actual experiment. Specifically, we compared the conventional analysis procedure using Agilent software to our calibration approach using a given CNV region in the mouse genome. The CNV analysed consisted of an approximate 5 kb fragment present in variable copy numbers between wild type individuals, but only one copy in the reference strain (C57B1/6). Figure 6 shows that the Agilent ratio analysis (upper panel) is indeed much noisier since many of the probes show values that are two to three standard deviations away from the average mean ratio (red and blue dots). In contrast, the calibrated probes (lower panel) showed mostly a smooth distribution. Both methods detected the CNV in question (indicated by the blue bar at the bottom), but the copy number estimate is expected to be more reliable for the calibrated probes. This comparison suggests that our protocol can be expected to result in fewer false positive calls and a better measuring capacity in CNV studies.

### Number of replicated probes

**Figure 7:**
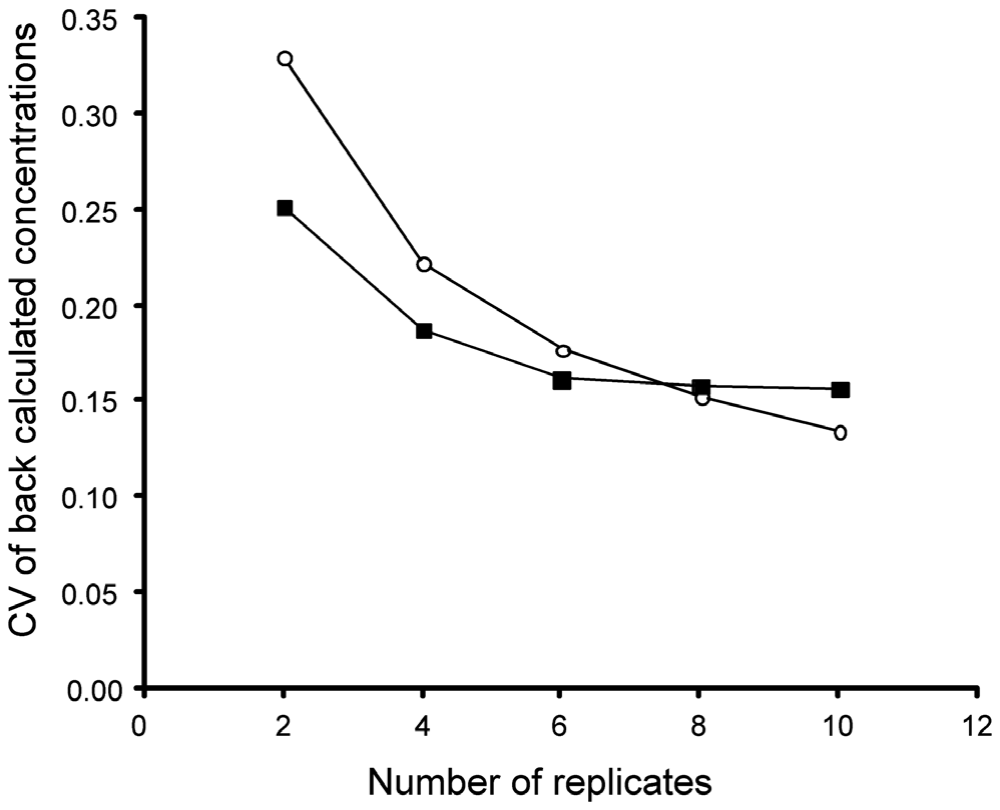
Coefficient of variation (CV) decrease for estimating the true concentration in dependence of probe replication. CV averages are displayed, circles, 25

Although the above experiments used 10 replicated probes for averaging, it would be of interest to know whether this is an optimal number. To address this question, we randomly selected 2 to 10 replicated probes from both 25 mer and 60 mer arrays and back-calculated the expected concentration of targets for the standard experiments (target concentration of 1×). The calculation was based on the calibration equations and parameters derived from 10 replicated probes because they are closest to the truth. As to be expeccted, we find a higher variance for estimating the true concentration when fewer probes were used (Figure 7). For the 25 mer array, 10 probes produced the lowest variance, but the shape of the curve suggests that even more probes might be beneficial. For the 60 mer array, we see no improvement beyond 6 replicates, i.e., this might be the optimal number of probes for this array type (Figure 7).

### Variance in the sample preparation

**Figure 8:**
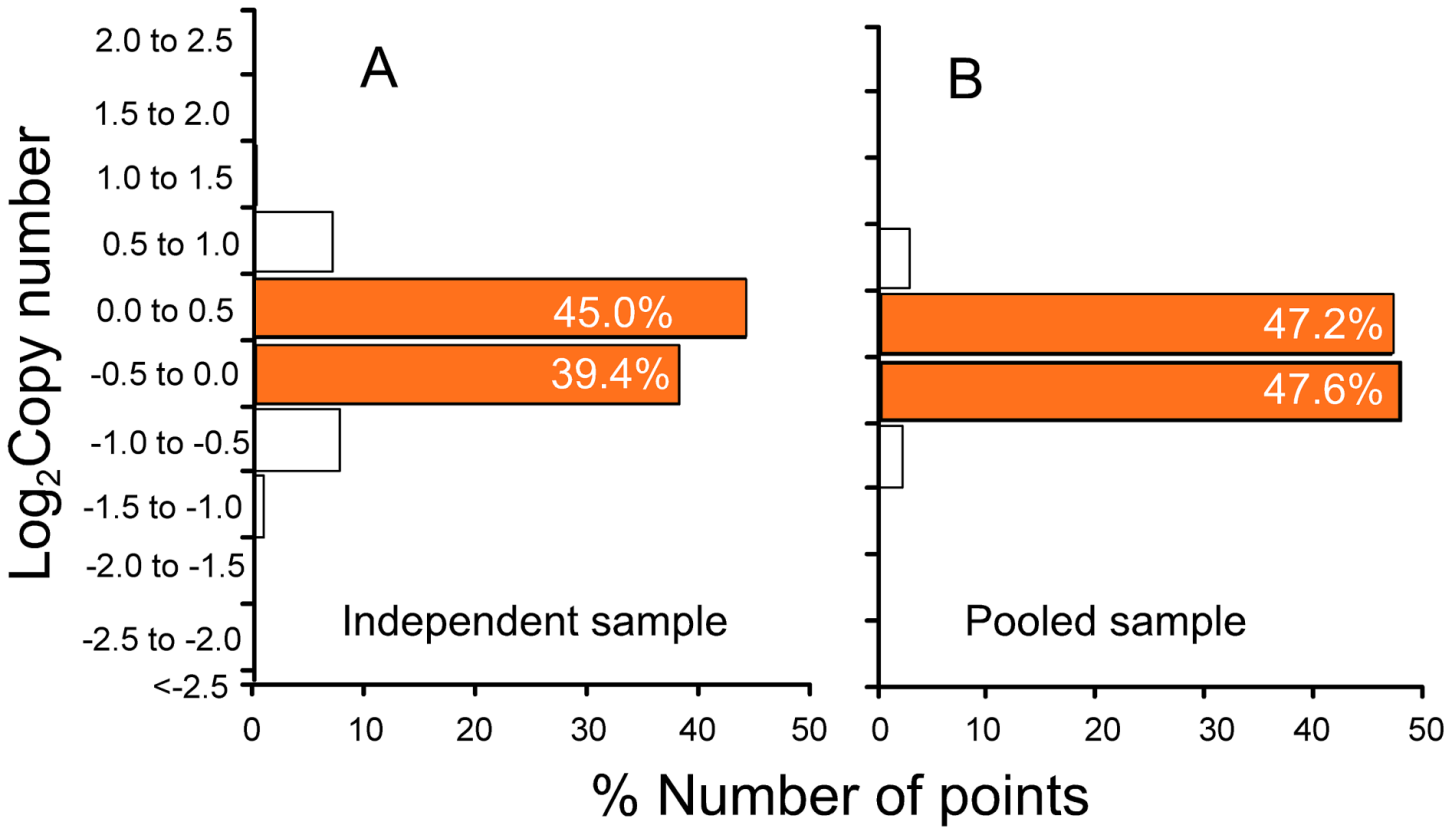
Comparison of probe labeling protocols on noise reduction. (A) Sample $ (*n* = 4,775) and (B) sample * (*n* = 4,767) from Figure 2. Note that the calibration was conducted without sample *. Ratios were calculated from calibration curves (R^2^>0.98) and a value is included into the histogram only if its relative error was under 20%.

Although the averaging and calibration removed much of the noise, a known additional source of noise comes from target preparation. Specifically, the target fragmentation and labelling procedures involve several enzymatic steps (i.e., PCR enzymatic digestion), which have previously been reported to introduce variability [30]. Figure 8 shows that the noise is indeed higher in the test sample (16.1% of comparisons outside the acceptable range) than in the pooled sample (5% outside). This result supports the notion that preparing multiple independent samples and then pooling the preparations can further reduce the variance inherent in the target sample preparation.

## Discussion

Given the broad application of microarrays in biological research and their success in determining gene expression patterns and structural genomic variation, one might ask: *why implement a new experimental procedure?* However, it has long been known that array results must be verified by an independent method, such as quantitative PCR, because they cannot be fully depended on. This inadequacy has lead some scientists to allude to the “end of arrays” (e.g., [31] and the substitution of array experiments with ‘next-generation’ sequencing since the latter is believed to be more reliable. Although sequencing based approaches have certain advantages compared to microarrays, the physicochemistry of neither technology has been well established [27]. We suggest that the utility of arrays has not been fully explored yet and the implementation of a new procedure that improves the interpretation of array output could be beneficial.

The results presented above may be considered as a proof of principle that assessing experimental noise and calibration can indeed improve microarray output. It will evidently be necessary to do large scale comparative experiments to fully assess the possible impact. The calibration procedure was already applied in one experimental study and did indeed yield a much better resolution of signals to allow clearer biological conclusions [32].

Our procedure is generally based on a common approach used in physics and analytical chemistry to experimentally determine the performance of a sensor (i.e., probe) and the magnitude of a measurement error. Once the measurement error is known, simple statistics can be used to obtain estimates of the true values. Knowing the error distribution, one can also reject outliers. We have conjectured and experimentally verified that there is indeed such an error distribution at the level of probe binding and target preparation (the labeling procedure). Our results show that the fidelity of estimating the true target concentration increased significantly with multiple replications of the probes and that this fidelity was dependent on probe length. Hence, we recommend that probe replication and averaging (steps 2 and 7 in Figure 1) should become a general standard, even if one uses otherwise the classical protocol. For 25 mer arrays, at least 10 probes seem to be necessary, while 6 probes seem to be sufficient for the 60 mer array. An evident drawback of replicating probes is that it limits the number of different probes that can be surveyed on an array. This presents a trade-off between the quality of signal and the number of different genes that can be studied, at least for array designs that can not compensate this with very high probe densities.

An additional element that we introduced into our revised design is the calibration of each individual probe (steps 4 and 8). It is often assumed that bioinformatic procedures for probe design are sufficient to optimize probe behavior. However, while some optimization is certainly possible in this way, it is evident that it does not fully solve the problem of huge differences in binding affinities between different probes (Figure S1 in File S1) and the possibility of non-linearity in the hybridization characteristics of probes. Thus, current array procedures use ratios of signals, based on comparisons with control targets, either on the same array using double labeling, or between arrays. But this has the drawback that the noise associated with the individual probe signal intensity (see above) is cumulative for sample and reference hybridization. Hence, control of this noise should be particularly important when a reference hybridization design is used. However, if probe-binding behaviors were known *a priori,* one can get an absolute quantification of signals. To achieve this, one needs a dose-response curve, which we have experimentally determined by recording the signals from a dilution series of the targets, followed by a fit to adsorption isotherm equations. Once an isotherm is known for a given probe, one can calculate the concentration of the target from the signal that is recorded. The derivation of the target concentration from the isotherm function takes care of non-linearity. Thus, a two-fold change, for example, means that the target concentrations in the samples that are compared differ indeed by exactly two-fold. In the current routinely used array algorithms, a two-fold change means only that signal intensities differ by two-fold, but with an unknown difference in the true target concentrations.

However, proper calibration is a challenge, since one needs to know the exact concentration of the target that is used for calibration. In case of calibration with a complex RNA sample, one does of course not know this and any measurement can therefore be only with the reference to the sample that was used for calibration, i.e. calibration yields only a small advantage over the normal reference hybridization procedure. The situation is a little bit better when gDNA is used for calibration, although it has also the uncertainty that the gDNA sample used may include regions that are subject to unknown CNV.

Using the biological sample itself for calibration entails also the risk that one is not only calibrating for the specific signal, but also for any unwanted nonspecific hybridization. The problem of cross-hybridization by similar target sequences can usually be addressed by applying algorithms in the probe design phase, provided full genome information is available. It remains a problem, though, that the total signal intensity contains specific and nonspecific hybridization signal and this will be probe-specific. Hence a remedy would be to design more than one probe for a given region (e.g., a gene) and compare the signals.

The best calibration would therefore be achieved with pure synthetic target DNA or RNA, but for arrays that are designed to record patterns from complex targets, this will evidently be very costly. Still, such an investment should be warranted for standard arrays, e.g., cancer research, hereditary diseases, etc., that are used in many experiments, since the data quality that can be obtained in this way would not require further verification experiments. We anticipate therefore that properly calibrated arrays will eventually become available. For the time being, one can use a well-defined target preparation for calibration.

## Supporting Information

File S1 **File includes Figures S1-S3 and Table S1.** Figure S1. Signal intensities of invariant probes on Affymetrix mouse genome diversity array. (A) Signal intensity dependence on GC content of the probes. (B) Signal intensity dependence on Gibbs free energy calculation of the probes. (C) Comparison of signal intensities of corresponding sense and antisense strands (R^2^ = 0.37). Figure S2. Comparisons of sense- and antisense stability of the same probes in solution and on the array. Probes (25 mers) were a subset from sense-antisense probes shown in Figure 1C. Corresponding targets were 98 nt oligonucleotides derived from the mouse genome sequence with the probe binding site in the middle of the target. (A) Melting temperatures of five sense-antisense pairs of probes. (B) Correspondence of melting temperatures to signal intensities on the array. Figure S3. Comparison of isotherms for a given probe (average values from 10 replicates). Left: hybridized to DNA (R2 = 0.99); right: hybridized to RNA (R2 = 0.99), the offset value c for this sample is ∼26. Table S1. Oligonucleotides used for the melting analysis in solution.
(DOC)

File S2 (ZIP)
