# Supplementary material for: A Revised Design for Microarray Experiments to Account for Experimental Noise and Uncertainty of Probe Response
Source: PLoS One. 2014 Mar 11;9(3):e91295. doi: 10.1371/journal.pone.0091295 (PMC3949741; doi:10.1371/journal.pone.0091295)
Supplement: File S1 — File includes Figures S1-S3 and Table S1. Figure S1. Signal intensities of invariant probes on Affymetrix mouse genome diversity array. (A) Signal intensity dependence on GC content of the probes. (B) Signal intensity dependence on Gibbs free energy calculation of the probes. (C) Comparison of signal intensities of corresponding sense and antisense strands (R2 = 0.37). Figure S2. Comparisons of sense- and antisense stability of the same probes in solution and on the array. Probes (25 mers) were a subset from sense-antisense probes shown in Figure 1C. Corresponding targets were 98 nt oligonucleotides derived from the mouse genome sequence with the probe binding site in the middle of the target. (A) Melting temperatures of five sense-antisense pairs of probes. (B) Correspondence of melting temperatures to signal intensities on the array. Figure S3. Comparison of isotherms for a given probe (average values from 10 replicates). Left: hybridized to DNA (R2 = 0.99); right: hybridized to RNA (R2 = 0.99), the offset value c for this sample is ∼26. Table S1. Oligonucleotides used for the melting analysis in solution. (DOC) [file pone.0091295.s001.doc]

# Supplementary MATERIAL

#
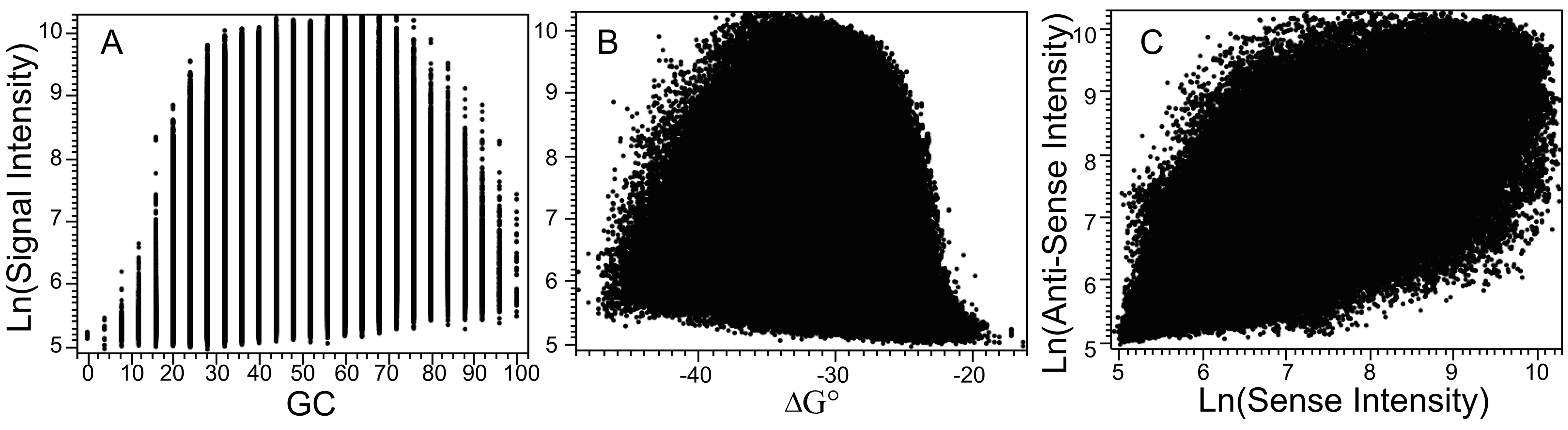


Figure S1. Signal intensities of invariant probes on Affymetrix mouse genome diversity array. (A) Signal intensity dependence on GC content of the probes. (B) Signal intensity dependence on Gibbs free energy calculation of the probes. (C) Comparison of signal intensities of corresponding sense and antisense strands (R^2^=0.37).


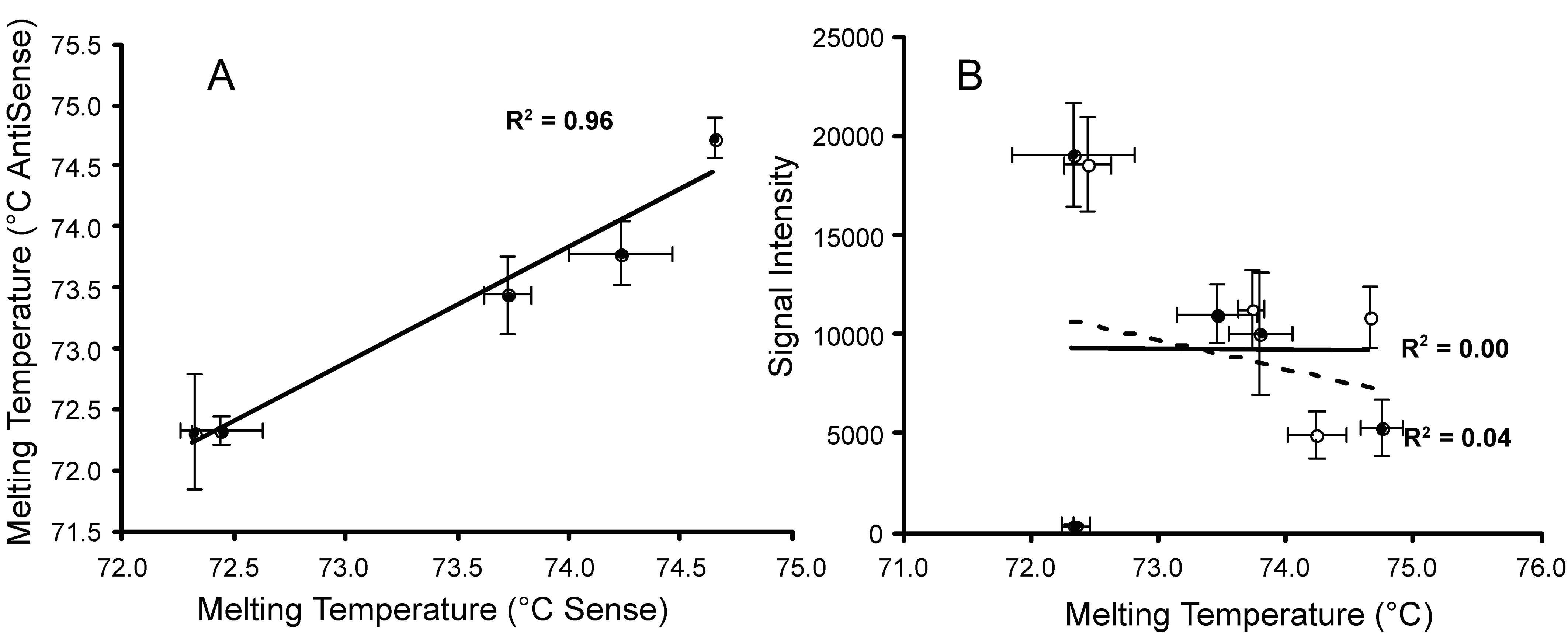


Figure S2. Comparisons of sense- and antisense stability of the same probes in solution and on the array. Probes (25mers) were a subset from sense-antisense probes shown in Figure 1C. Corresponding targets were 98 nt oligonucleotides derived from the mouse genome sequence with the probe binding site in the middle of the target. (A) Melting temperatures of five sense-antisense pairs of probes. (B) Correspondence of melting temperatures to signal intensities on the array.


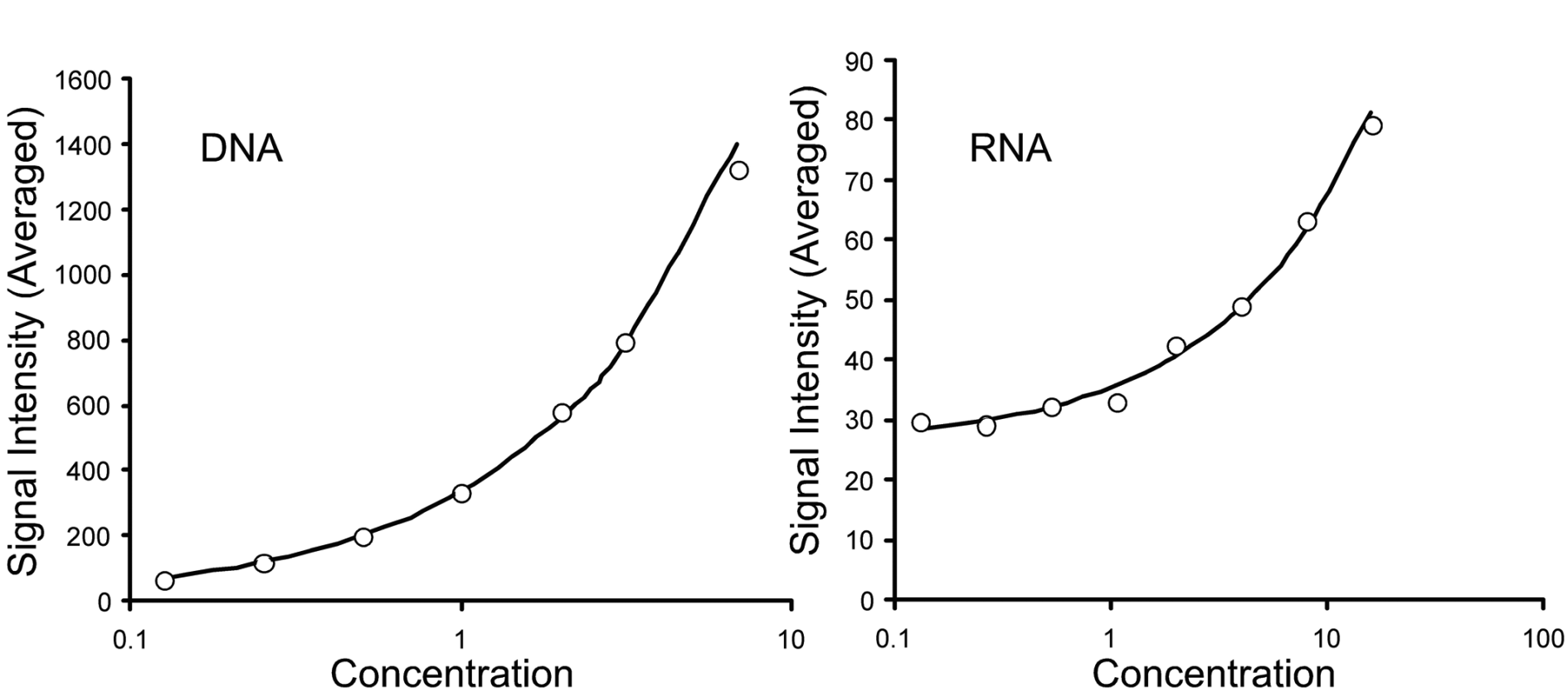


Figure S3. Comparison of isotherms for a given probe (average values from 10 replicates). Left: hybridized to DNA (R2=0.99); right: hybridized to RNA (R2=0.99), the offset value c for this sample is ~26.

# Methods

## Oligonucleotide probes and targets

The oligonucleotides involved in the melting experiments are listed in Table S1. Strands were paired as reg_NNNa (target) with NNNb (probe) and in the reverse order, reg_NNNb (target) with NNNa (probe). Five loci derived from the Affymetrix mouse genome diversity array (Affymetrix Corp.) were used to make the target sequences. The binding site for the probe was located in the middle of the target. Since both strands were represented, we made 10 target strands and 10 probes alltogether. The targets and probes were synthesized by Metabion International AG, Germany. DNA concentration was determined by the Beer–Lambert–Bouguer law from UV adsorption at 260 nm and the extinction coefficient (Table S1, right column).

## Melting experiments

The melting study was conducted in a buffer similar to the Affymetrix hybridization buffer. The Affymetrix hybridization buffer contains Denhardt’s solution, HSDNA (herring-sperm DNA), solution of control oligonucleotides, human Cot-1 DNA, Tween 20, DMSO, TMACL, MES and EDTA. HSDNA, Cot-1 DNA and control oligonucleotides relevant for array experiments. However, in our melting study some of these components would interfere with UV adsorption readings. Hence, they were replaced with corresponding volumes of water. The final buffer consisted of: MES 12x, 660 µl; EDTA 0.5 M, 165 µl; Tween-20 3%, 55 µl; DMSO 100%, 715 µl; TMACl 5 M, 7.7 ml and H_2_0, 1155 µl. In accordance with Affymetrix protocols, 2280 µl of the buffer were mixed with 876 µl of aqueous DNA solution such that the final concentrations of all components are: MES; 0.548 X; EDTA, 0.006 M; Tween-20, 0.011%; DMSO, 4.943%; TMACl, 2.662 M; probe, 1 nM; and target, 1 nM.

The association and dissociation experiments were conducted in a temperature-controlled cuvette in the Jas.co V630 spectrophotometer equipped with a magnetic stirrer (JASCO, Germany). A base-line measurement was a mix of 2280 µl buffer with 876 µl H_2_O. Immediately after the nucleic acids were added to the buffer, association kinetics was measured at 18^o^C for 140 min (Figure S1). In all cases, 140 min was considered sufficient time for the complete association of the duplex, which was followed by recording of a melt curve. A typical melting profile and its second derivative are shown on Figure S2. A melting temperature was determined at zero-crossing of the second derivative, changing its sign from positive to negative. All duplexes showed low (around 30^o^C) and high (around 70^o^C) melting temperatures. The low melting temperature is a characteristic of the buffer, which was determined by conducting a mock melting experiment with the buffer and water. Therefore, the low melting temperature was not considered in further analyses.

**Table S1**. Oligonucleotides used for the melting analysis in solution.

| OligoID | Sequence | Extinction Coefficient |
| --- | --- | --- |
| FRAn001649084a | GAAAATTTTGGTCTAGCTGGAGACG | 289 |
| FRAn001649084b | CGTCTCCAGCTAGACCAAAATTTTC | 267 |
| FRAn015210754a | GAATGTTTCTGCTTTTTCACAGACT | 263 |
| FRAn015210754b | AGTCTGTGAAAAAGCAGAAACATTC | 303 |
| FRAn016726462a | GAAACCCCGTCCCTCTTAAGGAGCA | 270 |
| FRAn016726462b | TGCTCCTTAAGAGGGACGGGGTTTC | 270 |
| FRAs000521504a | GTCCAACTGTAAGGCATTTTCTCAA | 273 |
| FRAs000521504b | TTGAGAAAATGCCTTACAGTTGGAC | 288 |
| SEQa026808595a | TATACATCTCAGAGGCAGAGGGGCT | 285 |
| SEQa026808595b | AGCCCCTCTGCCTCTGAGATGTATA | 260 |
| reg_FRAn001649084a | CTCACAGTCTCTGAGACCATGGGCATGTTGGCGAGATGAAAATTTTGGTCTAGCTGGAGACGATCAGTCCAGCATTCTTGAACCCCGAATGTGTTGGT | 1077 |
| reg_FRAn001649084b | ACCAACACATTCGGGGTTCAAGAATGCTAGACTGATCGTCTCCAGCTAGACCAAAATTTTCATCTCGCCAACATGCCCATGGTCTCAGAGACTGTGAC | 1074 |
| reg_FRAn015210754a | ATGATCCTGTTCAGATACTTAATATGGGGGAAAGGGGGAATGTTTCTGCTTTTTCACAGACTGATGTAAGAGCATGCTAGCTTCCAATGTGACAGGCT | 1102 |
| reg_FRAn015210754b | AGCCTGTCACATTGGAAGCTAGCATGCTCTTACAGCAGTCTGTGAAAAAGCAGAAACATTCCCCCTTCCCCCCATATTAAGTATCTGAACAGGATCAT | 1083 |
| reg_FRAn016726462a | AAAAACAAGAGAGAGAAGCAAGAGAGAGAGAGAAAACGAAACCCCGTCCCTCTTAAGGAGCATTCTCCTTCGCCTCGGACGTGTCACTCCTTGATTGG | 1124 |
| reg_FRAn016726462b | CCAATCAAGGAGTGACACGTCCGAGGCGAAGGAGAATGCTCCTTAAGAGGGACGGGGTTTCGTTCTCTCTCTCTCTTGTCTCTCGCTCTCTCTCTTGC | 1023 |
| reg_FRAs000521504a | AGAAAGGAGCTTCAGTTGAAGAAATGCCTCCACGAGGTCCAACTGTAAGGCATTTTCTCAATTAGTGATCAAGGGGAAAAGGCCCTTTGTGGGTGGGA | 1126 |
| reg_FRAs000521504b | CCCACCCACAAGGAGCCTTTCCCCCTTGATCACTAATTGAGAAAATGCCTTACAGTTGGACCTCATGCAGGCATTTCCTCAACTGAAGCTCCTTTCTC | 1030 |
| reg_SEQa026808595a | TTCGTGTGTGTTAGCCACAGTAACAACAGCACTAACTTATACATCTCAGAGGCAGAGGGGCTGAAGTTCTCCCTCTCCTTGGAGAATGTGCTGTATTA | 1077 |
| reg_SEQa026808595b | TAATACAGCACATTCTCCAAGGAGAGGGAGAACTTCAGCCCCTCTGCCTCTGAGATGTATAAGTTAGTGCTGTTGTTACTGTGGCTAACACACACGAA | 1091 |
