# Supplementary material for: A Revised Design for Microarray Experiments to Account for Experimental Noise and Uncertainty of Probe Response
Source: PLoS One. 2014 Mar 11;9(3):e91295. doi: 10.1371/journal.pone.0091295 (PMC3949741; doi:10.1371/journal.pone.0091295)
Supplement: File S2 — (ZIP) [file pone.0091295.s002.zip › ProgramsAndExampleDataset/DocumentationSoftware.docx]

DNA-meter, a calibrated microarray

# Step 1: Average and merge

For each experiment, organize microarray signal intensities into a simple text file of two columns, ProbeID Intensity:

A_67_P05181479 184.6739

A_67_P20444947 300.5581

A_67_P04577152 6376.69

A_53_P127264 37.15217

... ...

Run **average.exe** to average signal intensities per probe, assign experiment ID and merge experiments into one file and calculate standard errors.

## Command

average file1.txt id1 file2.txt id2... > out.txt

id1, id2, etc are experiment IDs; for calibration use concentrations.

## Examples

A calibration curve produced from a dilution series experiment (example dataset archive).

average calib_0_0625.txt 0.0625 calib_1.txt 1 calib_2.txt 2 calib_0_5.txt 0.5 calib_0_25.txt 0.25 calib_0_125.txt 0.125 > calibration_curve.txt

Single experiment (exp1_1_1raw.txt)

average exp1_1_1raw.txt 1_1_1 > exp1_1_1avg.txt

# Step 2: Calibrate microarray

If the microarray is already calibrated, proceed to Step 3. Run **calibrate.exe** which will use merged averaged signal intensities of the calibration curve and will produce parameters of calibration. Calibration output format is ProbeID a b R2 CurveID:

A_53_P100398 0.000691116 0.00325183 0.997242 L

A_53_P100736 4.28337 0.296658 0.981139 F

A_53_P102504 0.000593089 0.00524655 0.998836 L

... ...

## Command

calibrate < calibration_curve_file.txt [-T] [-R2 {0.9}] [-freund {F}] [-linear {LN}] [-langmuir {LG}] > out.txt

Input file calibration_curve_file.txt is produced in step 1.

-T tabulated output for inspection elsewhere

-R2 {0.9} R2 cut off, e.g 0.9, provided by user

-freund {F} Freundlich calibration; output marker, e.g. F, provided by user

-linear {LN} Linear calibration; output marker, e.g. LN, provided by user

-langmuir {LG} Langmuir calibration; output marker, e.g. LG, provided by user

When more than one calibration curve suggested, the one with the highest R2 is saved into the output.

## Example

Input file is produced by the example of Step 1 (calibration_curve.txt)

calibrate < calibration_curve.txt -R2 0.98 -freund F -langmuir L > calibration.txt

# Step 3: calculate concentrations

Run **concentration.exe** using averaged signal intensities from Step 1 and calibration parameters from Step 2 to calculate concentrations and standard errors.

## Command

concentration < calibrated_probes_file.txt [-freund {F}] [-linear {LN}] [-langmuir {LG}] -intens sample1.txt sample2.txt... > out.txt

-freund {F} Freundlich calibration y=exp(a)*x^b; input marker, e.g. F, provided by user

-linear {LN} Linear calibration y = a+b*x; input marker, e.g. LN, provided by user

-langmuir {LG} Langmuir calibration y = Y_max*K*x/(1+K*x); input marker, e.g. LG, provided by user a = 1/(K*Y_max); b = 1/Y_max

## Example

Averaged signal intensities for an experiment are produced in the example of Step 1 (exp1_1_1avg.txt) and calibration parameters are produced in the example of Step 2 (calibration.txt). Intensities are calculated and saved into output (conc_1_1_1.txt).

concentration < calibration.txt –freund F -langmuir L -intens exp1_1_1avg.txt > conc_1_1_1.txt
